# Head-to-head comparison of V-A ECMO, Impella and ECPELLA in normal ovine hearts

**DOI:** 10.1038/s41598-025-06457-0

**Published:** 2025-07-01

**Authors:** Konstantin Yastrebov, Hugh S. Paterson, David H. Tian, Laurencie M. Brunel, Fiona C. Schnitzler, Lisa M. Partel, Mark Dennis, Paul G. Bannon

**Affiliations:** 1 Prince of Wales Hospital, Randwick, NSW 2031 Australia; 2 The University of Sydney, Sydney, NSW 2006 Australia; 3 The University of New South Wales, Sydney, NSW 2052 Australia; 4 Royal Prince Alfred Hospital, Sydney, NSW 2050 Australia; 5 The Baird Institute, Sydney, NSW 2050 Australia; 6 Westmead Hospital, Sydney, NSW 2145 Australia; 7 Department of Intensive Care, Prince of Wales Hospital, Sydney, NSW 2031 Australia

**Keywords:** Impella, ECMO, ECPELLA, Hemodynamics, Myocardial work, Myocardial oxygen consumption, Cardiovascular models, Cardiac device therapy, Acute coronary syndromes, Heart failure, Interventional cardiology, Heart failure, Experimental models of disease, Translational research

## Abstract

Temporary mechanical circulatory support (MCS), including veno-arterial extracorporeal membrane oxygenation (ECMO) and micro-axial pumps (Impella), is increasingly used in clinical practice for refractory circulatory failure. Complex physiological responses to each technique or their combination (ECPELLA) remain debated and are often specific to cardiovascular pathology. A paucity of data on physiological responses to MCS in normal subjects makes comprehensive understanding of such responses in variable disease states difficult, as well as during weaning MCS in recovering hearts. This translational investigation compared three MCS techniques with variable pump flows in healthy sheep (*n* = 7) to establish baseline for future studies in cardiomyopathic models. All MCS techniques increased arterial elastance, but reduced LV myocardial work, coronary arterial flow and LV myocardial oxygen consumption. ECPELLA was more effective in increasing total systemic blood flow and MAP. The overall similarity between the MCS techniques suggests that the more invasive and complex combination of devices (ECPELLA) can only be justified for management of the severe failing heart as the means for decompressing LV. A study investigating the comparative impacts of different regimes and MCS techniques in a cardiomyopathic model is warranted.

## Introduction

Temporary mechanical circulatory support (MCS) is increasingly used in clinical practice for patients with cardiogenic shock, refractory circulatory collapse, cardiac arrest, and for procedural support in operating theatres and cardiac catheterization laboratories^1^. Commonly used mechanical circulatory support devices include veno-arterial extracorporeal membrane oxygenation (ECMO) in central or peripheral configuration, and microaxial pumps (Impella)^2^.

Central ECMO is used with cardiac surgery when the chest is open and the heart cannot be safely weaned from cardiopulmonary bypass. Peripheral ECMO, where cannulation is most commonly of the femoral vessels, allows full or partial cardiovascular support as well as correction of inadequate gas exchange within the pulmonary circulation. ECMO provides bi-ventricular support (cardio-pulmonary bypass and oxygenation) with reportedly reduced LV stroke volume and stroke work and increased systemic mean arterial pressure (MAP)^2^. The reported impact of ECMO on left ventricular end-diastolic pressures (LVEDP) is controversial^3,4^. Despite a decrease in systolic blood pressure (SBP) with ECMO, there is an increase in the systemic afterload which is commonly taught to have the potential to impede a very severely impaired LV from ejecting adequately^5^ leading to increased myocardial work, raised LVEDP and LV dilatation^6–8^. The failure of ejection can cause LV decompensation beyond the maximal pressure response to filling, as seen on the failure end of the Frank-Starling curve, although this is not completely supported in recent human work^8^. The overall impact of ECMO on survival of patients with cardiogenic shock and cardiac arrest remains debated^9^.

The Impella™ is a micro-axial pump that traverses the aortic valve, pumping blood directly from the LV into the aorta. It is used as a temporary left ventricular assist device^10^ reducing LV work and end-diastolic pressure (LVEDP) and increasing MAP in clinically failing hearts^11^. Right ventricular support is mostly limited to the ventricular interaction with reduced postcapillary pulmonary hypertension. The overall impact of an Impella on the survival of patients with cardiogenic shock from acute myocardial infarction (AMI) had been controversial until the recent DanGer Shock clinical trial^12^which suggested a survival benefit in carefully selected patients, albeit with increased morbidity.

ECPELLA (also referred to as ECMELLA) – is a circulatory support technique which simultaneously combines ECMO and Impella^13^. The addition of an Impella to ECMO support ensures adequate decompression of very dysfunctional LV that cannot eject against the systemic input impedance, further elevated by ECMO^6^. Limited observational studies have reported a mortality benefit in patients with cardiogenic shock and cardiac arrest^13^. The impact of the flow settings of each device (Impella and ECMO) when using ECPELLA is not clear. The impact of these MCS techniques on normal or recovering heart is unknown.

### Hypothesis

Circulatory responses and myocardial oxygen consumption may differ during application of various regimes and different MCS techniques to the normal or recovering heart.

### The aim of investigation

To determine potential differences in physiological sequalae associated with application of variable flow rates of ECMO, Impella and ECPELLA, including global and cardiac haemodynamic effects as well as myocardial oxygen consumption in normal sheep.

## Results

A total of 229 datapoints were recorded for analysis at variable mechanical circulatory support flows (81 data points for Impella, 82 data points for V-A ECMO, 66 data points for ECPELLA). These datapoints were based on zero flow datapoints plus datapoints at increased MCS flows performed progressively in five steps for ECMO and Impella, and zero flow plus four steps for ECPELLA, until reaching maximum possible flows as the final datapoint in each episode of MCS application. Separate datapoints were recorded during two similar runs for each MCS device in each sheep. Missing data points reflect episodes of maximum flow achieved before the last point of the run. The directly measured haemodynamic and flow parameters in relation to MCS flows are presented in Table 1. The calculated parameters (as defined below) are presented in Table 2. The probability of different responses of each parameter to each MCS device comparison is presented in Table 3. In the comparison of Impella and ECMO, the differences in CVP and PA flow reflect the difference in device blood drainage flow. The comparison of ECMO and ECPELLA reflects greater venous return to the right heart in ECPELLA during equal total MCS flows.

**Table 1:** Directly measured parameters.

Haemodynamic parameters					
**Parameter**	**MCS**	**Baseline**	**Highest flow**	**β (95% CI) mmHg/L MCS**	**P value**
Heart rate*	Impella	92.6 ± 8.1	89.2 ± 6.0	-0.28 (-1.65–1.10)	0.697
	ECMO	88.0 ± 9.4	92.8 ± 10.4	0.56 (-0.84–1.97)	0.429
	ECPELLA	89.8 ± 7.9	86.6 ± 8.0	-0.34 (-1.25–0.57)	0.460
Systolic aortic BP^	Impella	107.2 ± 19.0	86.8 ± 15.1	-6.39 (-9.58 - -3.25)	< 0.001
	ECMO	107.2 ± 17.9	100.2 ± 24.2	-3.68 (-6.88 - -0.48)	0.024
	ECPELLA	103.8 ± 20.7	103.5 ± 26.5	0.73 (-1.35–2.80)	0.491
Diastolc aortic BP^	Impella	76.8 ± 18.2	72.7 ± 15.0	-1.58 (-4.55–1.36)	0.300
	ECMO	76.4 ± 16.6	79.2 ± 23.4	-0.21 (-3.20–2.78)	0.891
	ECPELLA	73.9 ± 20.0	95.5 ± 26.1	4.03 (2.09–5.97)	< 0.001
Mean aortic BP^	Impella	91.8 ± 18.5	77.0 ± 15.6	-4.62 (-7.61 - -1.66)	0.003
	ECMO	90.7 ± 17.0	88.5 ± 24.2	-1.93 (-4.97–1.10)	0.210
	ECPELLA	88.9 ± 18.7	97.1 ± 22.4	2.39 (0.43–4.35)	0.017
Pulse pressure^	Impella	30.4 ± 10.1	13.0 ± 6.9	-4.93 (-6.44 - -3.41)	< 0.001
	ECMO	30.8 ± 9.0	21.1 ± 5.6	-3.43 (-4.98 - -1.88)	< 0.001
	ECPELLA	29.9 ± 12.6	7.4 ± 4.1	-3.37 (-4.37 - -2.37)	< 0.001
Central venous pressure^	Impella	11.9 ± 3.6	10.1 ± 2.9	-0.66 (-1.06 - -0.26)	0.002
	ECMO	13.3 ± 3.5	7.5 ± 3.8	-1.85 (-2.26 - -1.44)	< 0.001
	ECPELLA	12.4 ± 3.1	8.4 ± 3.2	-0.66 (-0.93 - -0.40)	< 0.001
Left atrial Pressure^	Impella	15.5 ± 5.1	9.1 ± 4.5	-1.57 (-2.28 - -0.87)	< 0.001
	ECMO	15.9 ± 4.6	7.3 ± 4.4	-2.16 (-2.85 - -1.47)	< 0.001
	ECPELLA	15.7 ± 5.1	7.3 ± 3.8	-1.39 (-1.84 - -0.94)	< 0.001
**Flow parameters (ml/Min)**					
Main pulmonary artery	Impella	4.211 ± 1.311	3.786 ± 0.772	-0.078 (-0.194–0.038)	0.187
	ECMO	4.243 ± 1.198	1.257 ± 0.761	-0.970 (-1.086 - -0.853)	< 0.001
	ECPELLA	4.129 ± 1.290	2.293 ± 0.732	-0.299 (-0.374 - -0.224)	< 0.001
Left main coronary artery	Impella	166.1 ± 49.9	113.4 ± 43.0	-15.58 (-21.88 - -9.18)	< 0.001
	ECMO	158.6 ± 53.7	110.6 ± 39.8	-18.21 (-24.55 - -11.87)	< 0.001
	ECPELLA	157.3 ± 47.3	100.5 ± 24.8	-10.40 (-14.50 - -6.29)	< 0.001
**Haemoglobin oxygen saturation %**					
Coronary venous	Impella	50.5 ± 16.3	61.0 ± 17.3	3.68 (1.54–5.82)	0.001
	ECMO	60.1 ± 13.2	67.9 ± 11.1	3.75 (1.71–5.80)	< 0.001
	ECPELLA	57.1 ± 13.9	72.1 ± 15.9	2.63 (1.29–3.97)	< 0.001

* Beats per minute.

^ mmHg.

**Table 2:** Calculated Parameters.

Parameter	MCS	Baseline	Highest flow	β (95% CI) unit/L MCS flow	*P* value
LV stroke volume (ml)	Impella	45.7 ± 18.0	6.9 ± 6.5	-11.63 (-13.07 - -10.18)	< 0.001
	ECMO	47.6 ± 17.2	14.2 ± 9.2	-10.93 (-12.40 - -9.46)	< 0.001
	ECPELLA	44.1 ± 16.3	-3.3 ± 5.0	-8.52 (-9.47 - -7.57)	< 0.001
LV stroke work SAP (ml*mmHg)	Impella	3945 (3500–4502)	540 (142–849)	-1140 (-1346 - -931)	< 0.001
	ECMO	3514 (3437–4417)	1122 (600–1784)	-1027 (-1238 - -816)	< 0.001
	ECPELLA	4073 (3272–6196)	-216 (-391 - -27)	-926 (-1062 - -789)	< 0.001
LV stroke work MAP (ml*mmHg)	Impella	3945 (3500–4502)	540 (142–849)	-1140 (-1346 - -931)	< 0.001
	ECMO	3514 (3437–4417)	1122 (600–1784)	-1027 (-1238 - -816)	< 0.001
	ECPELLA	4073 (3272–6196)	-216 (-391 - -27)	-926 (-1062 - -789)	< 0.001
LV myocardial O_2_ consumption (ml/min)	Impella	7.5 ± 3.1	3.7 ± 2.9	-1.12 (-1.50 - -0.73)	< 0.001
	ECMO	5.7 ± 2.8	2.9 ± 1.6	-1.24 (-1.60 - -0.87)	< 0.001
	ECPELLA	5.0 ± 2.3	1.9 ± 1.2	-0.59 (-0.83 - -0.34)	< 0.001
Total systemic blood flow (TSBF) (ml/min)	Impella	4.2 ± 1.0	3.8 ± 0.8	-0.08 (-0.19–0.04)	0.189
	ECMO	4.2 ± 0.9	4.4 ± 0.9	0.03 (-0.09–0.15)	0.607
	ECPELLA	4.1 ± 1.0	4.8 ± 1.1	0.20 (0.12–0.27)	< 0.001
Ea (mmHg/ml)	Impella	1.9 (1.8–2.3)	9.7 (6.8–20.3)	3.01 (1.38–4.64)	< 0.001
	ECMO	1.9 (1.6–2.0)	5.3 (4.4–9.4)	2.40 (0.83–3.96)	0.003
	ECPELLA	1.7 (1.7–2.2)	22.4 (12.9–23.2)	3.48 (2.28–4.68)	< 0.001
SVR (mmHg*min/L)	Impella	1415 (1363–1569)	1351 (1093–1444)	-43 (-93–8)	0.100
	ECMO	1352 (1172–1578)	1411 (1184–1707)	-20 (-71–31)	0.446
	ECPELLA	1311 (1233–1702)	1309 (1129–1579)	-4 (-37–29)	0.830

*SVR*  systemic vascular resistance. *Ea*  arterial elastance.

Increases in Impella, ECMO or ECPELLA flows did not result in changes in heart rate. (Table 1).

**Fig 1:**
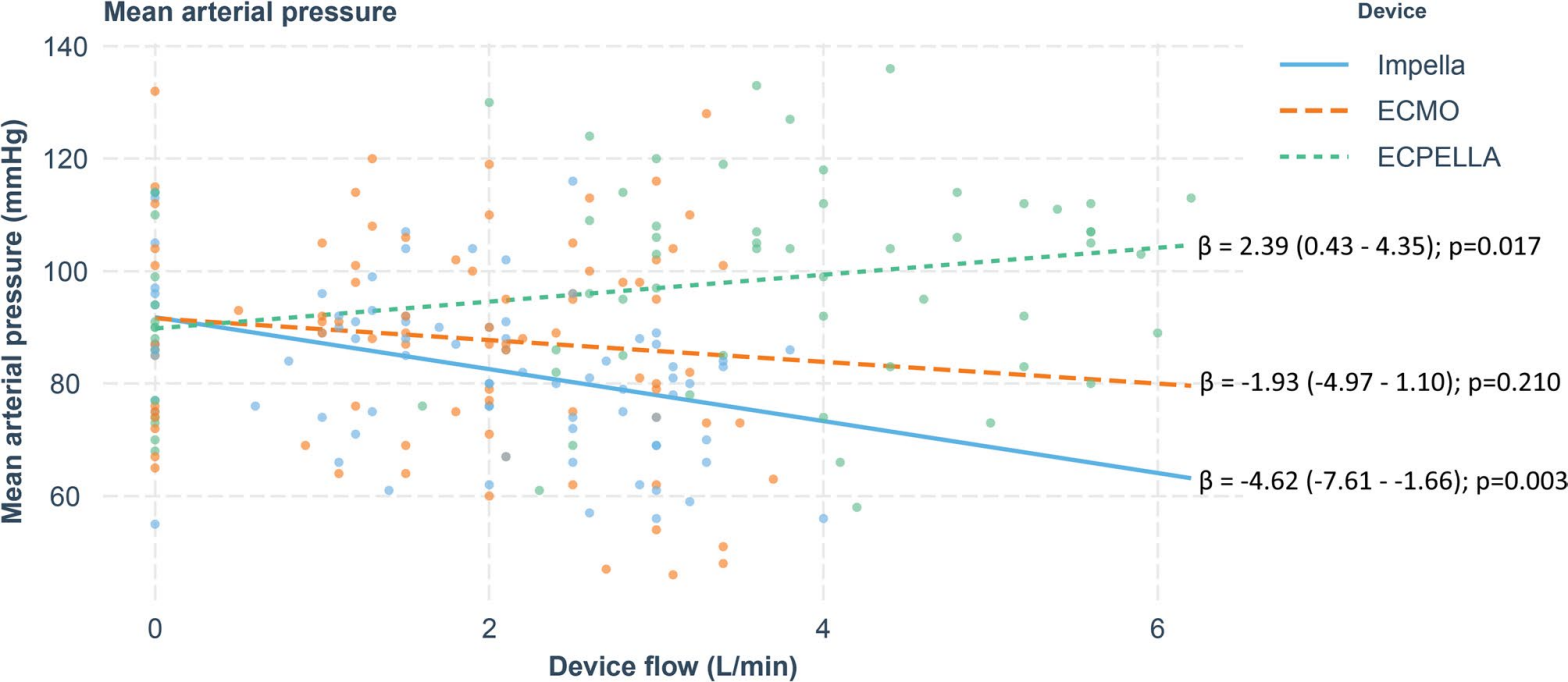
Mean arterial pressure and mechanical circulatory support flows. Betta-coefficient indicates changes in MAP in mmHg per each 1 l/min increase in MCS flow. Values in brackets indicate 95% confidence interval. MAP was slightly reduced by Impella, remains unchanged with ECMO and was slightly increased by ECPELLA.

Increasing Impella and V-A ECMO flows resulted in a decrease in SBP. Increasing ECPELLA flow did not result in significant changes in SBP (Table 1). Increasing Impella and V-A ECMO flows did not result in changes in DBP. Increase in ECPELLA flows resulted in an increase in DBP (Table 1). Increasing Impella flow resulted in a decrease in MAP. There were no changes in MAP with increased ECMO flows. Increase in ECPELLA flows resulted in an increase in MAP (Table 1; Fig. 1). Pulse pressure consistently decreased with increasing flows of all circulatory support devices (Table 1). CVP was reduced with increasing flows of all three circulatory support techniques (Table 1). CVP decreased with ECMO, which was nearly three times more effective than Impella and ECPELLA. Left Atrial Pressure was equally reduced with increased flows of all circulatory support devices (Table 1). Increasing Impella and ECMO flows did not result in changes in TSBF. Increase in ECPELLA flow resulted in an increase in TSBF (Tables 1 and 3). Increasing Impella flows did not result in changes in native right ventricular cardiac output, while ECMO and ECPELLA decreased it. (Table 1). Increasing flows of all circulatory support devices resulted in a significant decrease in native left ventricular stroke volume, with ECPELLA having the least effect (Table 1).

**Table 3:** P values for changes in parameter between different devices.

	ECMO v Impella	ECMO v ECPELLA	Impella v ECPELLA
Systolic blood pressure	0.240	0.042	**< 0.001**
Diastolic blood pressure	0.503	0.041	**0.001**
Mean arterial pressure	0.234	0.036	**< 0.001**
Pulse pressure	0.021	0.993	0.158
Central venous pressure	**< 0.001**	**< 0.001**	0.866
Left atrial pressure	0.274	0.054	0.251
Total systemic blood flow	0.172	0.017	**< 0.001**
Native right ventricular output	**< 0.001**	**< 0.001**	0.003
Native left ventricular stroke volume	0.452	0.008	0.002
Left ventricular oxygen consumption	0.656	0.003	0.017
Coronary artery flow	0.582	0.091	0.018
Stroke work	0.410	0.513	0.124

* bold represents significant when adjusted for multiple testing.

### LV native stroke work

**Fig 2:**
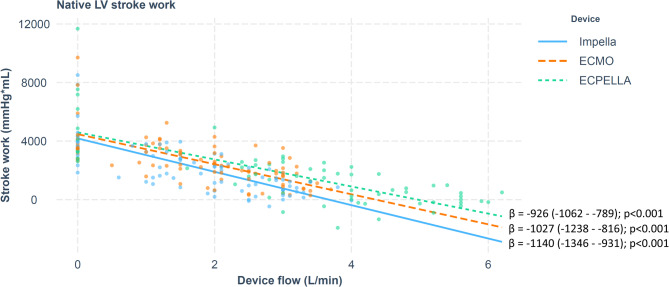
Left ventricular stroke work and mechanical circulatory support flows. Betta-coefficient indicates changes in LV SW in ml*mmHg per each 1 l/min increase in MCS flow. Values in brackets indicate 95% confidence interval. All MCS techniques reduced LV SW, with ECPELLA being the least efficient.

Increasing flows by all circulatory support devices resulted in decreases in LV native stroke work (Tables 1 and 3; Fig. 2).

### Left main coronary artery flow

**Fig 3:**
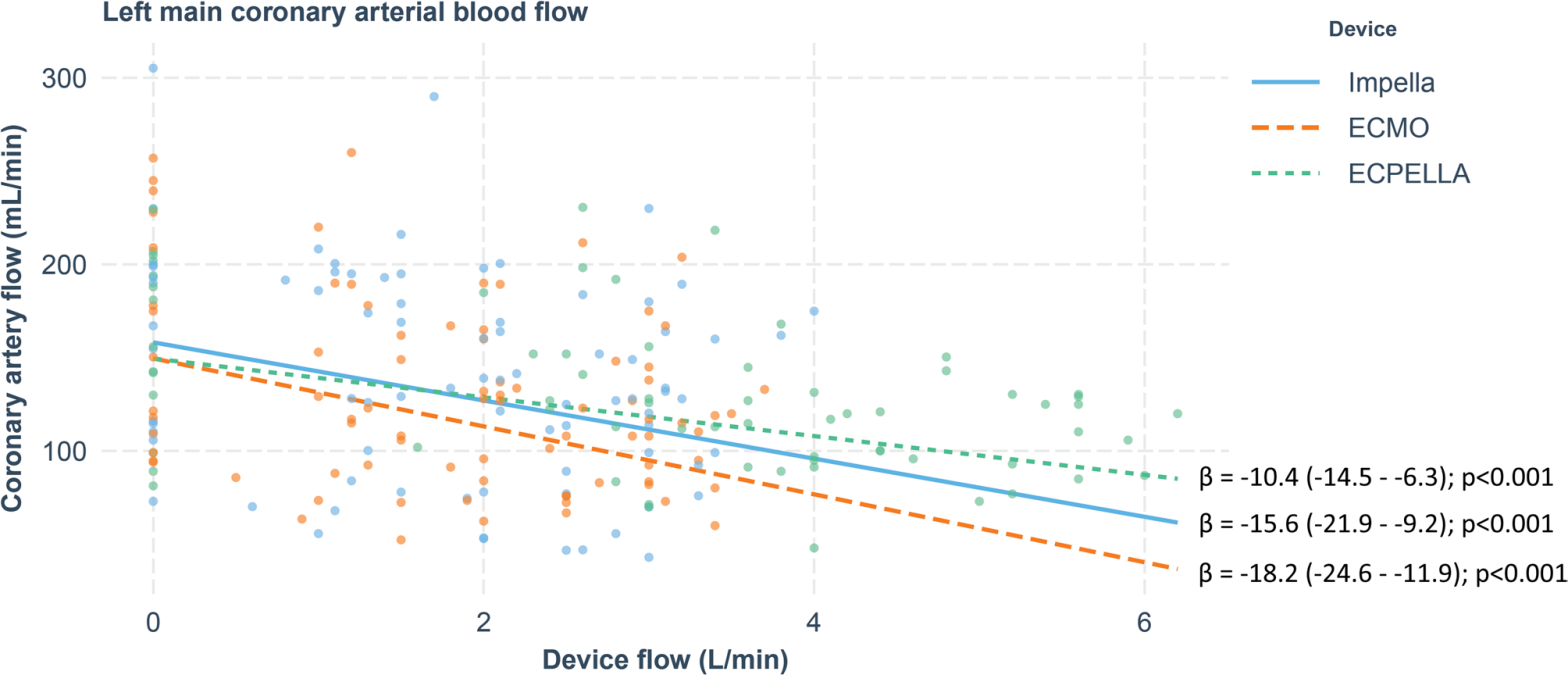
Coronary arterial flow and mechanical circulatory support flows. Betta-coefficient indicates changes in coronary flow in ml/min per each 1 l/min increase in MCS flow. Values in brackets indicate 95% confidence interval. All MCS techniques reduced coronary arterial flow, with ECPELLA having the least impact.

Increasing flow of all MCS devices resulted in decreased coronary arterial flow (Table 1; Fig. 3). The overall stability of DBP with increasing flows of the three MCS could suggest that changes in coronary artery flow were independent from changes in DBP. However, when the analysis was performed with stratification by individual animal and experimental runs, there was significant dependent relationship between DBP and coronary flow in all MCS techniques. Betta coefficients were 1.39 (95% CI 0.74–2.03, p˂0.001) for Impella, 1.52 (95% CI 1.08–1.96, p˂0.001) for ECMO and 0.52 (95% CI 0.13–0.91, *p* = 0.01) for ECPELLA.

### Coronary sinus hemoglobin oxygen saturation (SvO_2_)

Oxygen saturation of hemoglobin in the coronary sinus increased nearly equally with increasing flows from all circulatory support devices (Table 1).

### LV myocardial oxygen consumption

**Fig 4:**
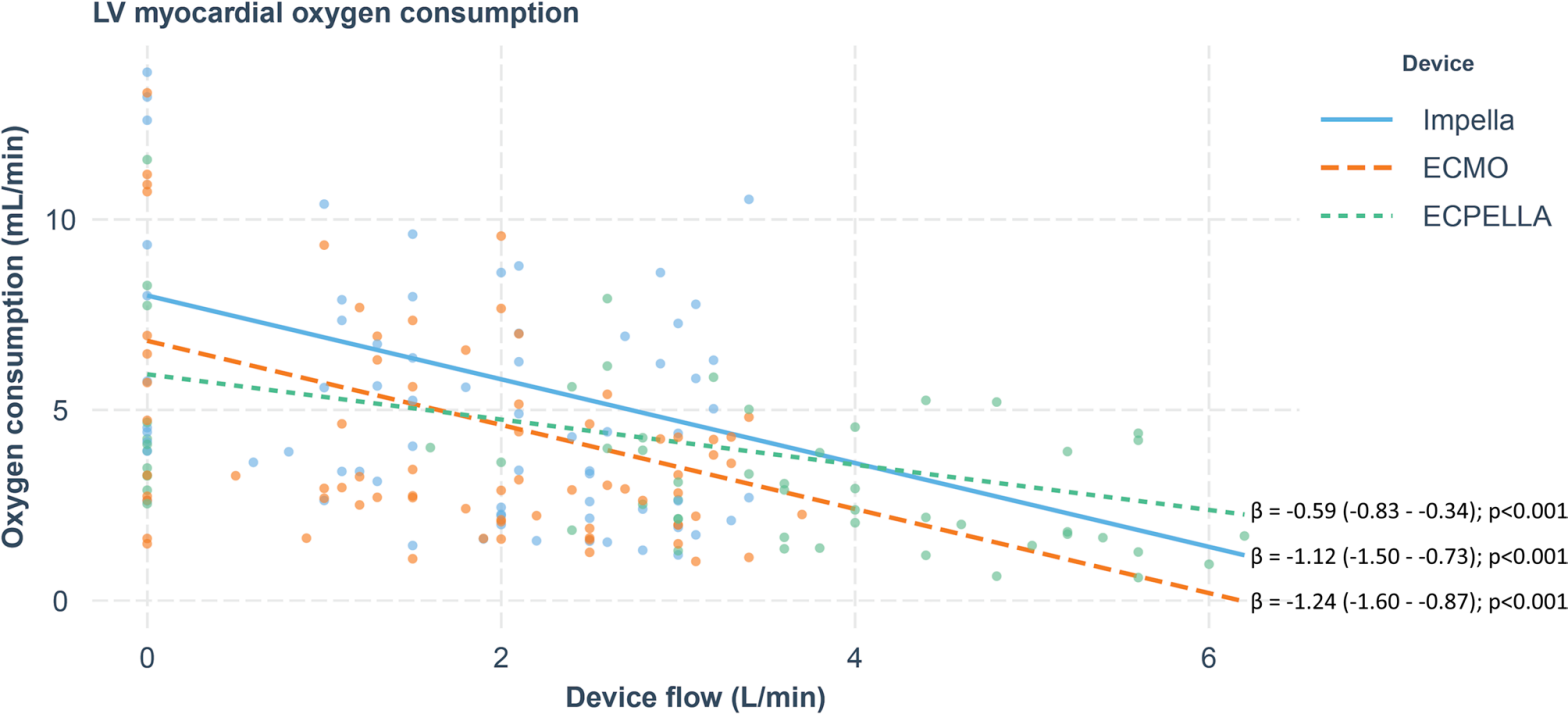
Myocardial oxygen consumption and mechanical circulatory support flows. Betta-coefficient indicates changes in myocardial oxygen consumption in ml/min per each 1 l/min increase in MCS flow. Values in brackets indicate 95% confidence interval. All MCS techniques reduced myocardial oxygen consumption with ECPELLA being the least efficient.

Left ventricular myocardial oxygen consumption decreased with increasing flows in all devices. LV stroke work, myocardial oxygen consumption, coronary flow, and oxygen extraction from the blood (SaO2-SvO2) all decreased with increasing MCS flows with no significant difference between devices (Tables 1 and 3; Fig. 4).

### Arterial elastance

A consistent increase in arterial elastance was found with increasing flows of all MCS devices.

### Echocardiography

Mild aortic incompetence was detected in all animals following implantation of the Impella. Echocardiographic monitoring did not demonstrate aortic valve incompetence beyond mild grade at any time during conducted experiments.

#### Limitations

#### Pressure-volume loops

Increasing MCS flows decreased LV volumes, eventually resulting in interference between myocardial walls, the Impella and the LV conductance catheter such that reliable pressure-volume data could not be obtained at the higher flows.

#### Impella flow measurement

Physiologically implausible negative native LV output was calculated at several data points during maximally achievable Impella and ECPELLA support flows. Impella flows were recorded from the display of the Impella controller device. The power required to generate the flow was calibrated and standardized in the laboratory conditions to the generated flows by the device developers. Displayed flows are being calculated based on the measured device power. We calculated native LV cardiac output as the difference between main pulmonary artery flow and displayed by the Impella controller flow. Therefore, the negative values reported from our experiments most likely resulted from falsely high Impella flows displayed by the controller during maximum flows. Importantly, such negative values were only seen with high Impella flows and predominantly with ECPELLA high flows. When the ventricle is emptied by MCS to a very low residual volume, a partial “suck-in” effect could increase the energy used by the motor and therefore could cause an overestimation of the flow rate at high Impella flows. In the absence of echocardiographic evidence for any significant aortic incompetence, we concluded that the Impella console overestimated the flow when the LV was nearly empty. This is unlikely to be an issue in a cardiomyopathic heart but should be considered in patients with small hypertrophic ventricles during myocardial recovery.

## Discussion

MCS is increasingly used in clinical practice to support a failing native circulation (cardiogenic shock) unresponsive to pharmacological therapy alone^14^. Despite technological improvements and growing worldwide clinical experience, mortality among patients requiring MCS remains high^15^outcome benefits uncertain^12^ and physiological implications complex^2^. This translational investigation was designed to investigate the comparative influence of three MCS techniques (Impella, Central ECMO and ECPELLA) with variable pump flows on hemodynamic parameters and myocardial oxygen consumption in normal sheep. Whilst it may be assumed that responses would be different in cases of cardiogenic shock, no comparative studies have been reported to enable better understanding of physiological responses to the specifics of each MCS technique in normal hearts, to allow better comprehension of human responses, particularly during recovery from cardiomyopathic conditions. The findings of this study could shine the new light on complex haemodynamic responses to MCS, offering optimized approach to MCS weaning during the recovery phase in cardiomyopathic hearts undergoing MCS support.

Previous experimental and clinical hemodynamic data suggested the usual increase in systolic and in mean arterial pressure following initiation of MCS^7^although ECMO was also previously shown in human data to have SAP and MAP unchanged with increased flows^8^. Our results surprisingly demonstrated a consistent reduction in SBP when using Impella and ECMO individually, while unchanged in ECPELLA. Impella also reduced MAP, while MAP increased with ECPELLA. Only ECPELLA increased DBP, which remained stable with other MCS. The decreases in SBP can be mostly explained by reduced pulsatility during increasing continuous MCS flows. ECMO did not change MAP and the total systemic blood flow, while Impella reduced MAP and ECPELLA increased it. Calculation of MAP in presence of unchanged DBP but reduced SBP due to the diminished pulsatility, explains the results for Impella. Conversely, the increase in DBP with unchanged SBP explains the result for ECPELLA. The important discrepancy of MAP changes derived from clinical data and this study during MCS support, is likely a result of our model with normal hearts. MCS is employed in severely failing hearts in clinical practice. Those patients are positioned on the flat or even receding portion of the Frank-Starling curve. In contrast, our animals had normal hearts, being at the steep portion of the curve, where reduction in LV filling induced by MCS, generate the most decrease in contractile response.

MCS is used when organ perfusion is deficient. End organ perfusion is dependent on both MAP and CO and a deficiency of either or both can be corrected with MCS to improve organ perfusion. In cardiomyopathy, the MCS is an additive to the total blood flow. In our normal heart ovine model, there were only modest changes in MAP and total systemic flow suggesting the MCS replaced cardiac function without adding to it. This should be the underlying principle to explain the changes seen in this study.

Our results suggested a consistent increase in arterial elastance during accelerating mechanical support (Table 2). Increase in arterial elastance have been previously reported during clinical investigations of ECMO^8^animal experiments of LV unloading with Impella while concurrently using noradrenaline in profound cardiogenic shock^16^and in animal research of haemodynamic effects of ECPELLA during cardiogenic shock^17^. Analysis of Ea from pressure-volume loops during application of Impella is complicated by progressive triangulation of the loops with increasing pump flows, which probably explains nuanced evidence, particularly from the simulation studies. We postulate that the increase in Ea found in our experiments with all MCS devices is likely a result of mechanical redistribution of extra volume from the venous to the arterial compartment by MCS pumps with simultaneous reduction in LV SV due to the reduced LV end-diastolic volume. Noradrenaline was used in stable dosage during experiments which could also compound to the results. We recorded the trend to the reduction in traditionally calculated SVR, although it did not reach statistical significance. The finding is hypothesis-generating that an intricate change of total systemic impedance in presence of increased elastance could be caused by compensatory arterial vasodilation to enable an adequate outflow to preserve venous return.

The decrease in native right ventricular output was physiologically expected with reduction in RV preload induced by the drainage of the venous blood by application of ECMO and ECPELLA. Impella had no significant impact on native RV output, which is consistent with the “Guytonian” principles of circulation (CO = systemic venous return)^18^ and inherently different device functions. All three techniques significantly reduced native LV stroke volume due to the reduction in LV diastolic filling.

There was a significant and consistent decrease in the left main coronary arterial flow induced by all three MCS techniques and a parallel decrease in native LV stroke work, all contributing to decreased LV myocardial oxygen consumption and increase in coronary sinus oxygen content. Importantly, careful analysis of individual MCS runs in each sheep demonstrated significant correlation between DBP and coronary flow. The causation of this relationship could not be directly implied from our experimental design. It is expected that coronary reactivity and autoregulation, affecting coronary arterial flow, will be different in various disease states in clinical practice. Our results appeared to reflect a multi-level auto regulation according to supply and demand. While these results are in contrast with some previous studies^6^the reduction in LV SW and in myocardial oxygen consumption are consistent with previous suggestions that MCS may reduce both kinetic and potential energy expenditure by the myocardium with increased pump flows^8^.

This investigation used central ECMO, where the direction of return flow is antegrade. It is still widely accepted that retrograde flow used in a peripheral ECMO configuration may be responsible for increases in systemic input impedance^2^. However, Ohm’s Law and the Bernoulli Equation principles combined, used to explain native circulation, are insufficient to fully interpret the impact of MCS devices which are generating and transmitting additional energy associated with pump flows in continuous, rather than native (pulsatile), fashion. Investigations utilizing 3D computational fluid dynamics modelling suggested complex interactions between continuous peripheral ECMO flows and residual native pulsatile LV output^19^. The precise nature of the distribution and exchange of kinetic and potential energies within two counterflows and associated significant vortices is unknown. It could be also presumed that the impacts on cardiac work and myocardial oxygen consumption by Central vs. Peripheral ECMO could differ, particularly due to the expected difference in arterial blood column inertia and a difference in impacting reflective pulse waves in the arterial tree. Central ECMO use is uncommon and is essentially restricted to failure to wean from cardiopulmonary bypass after cardiac surgery. Even then, conversion to peripheral ECMO is usually preferred to allow closure of the sternotomy. Accordingly, the translational benefits of this study are dependent on a similar comparison of peripheral and central ECMO. Central ECMO was used in this study due to the limitations of ECMO application in quadrupeds with relatively small femoral vessels limiting peripheral ECMO flow rates.

The translational value of the study includes not only the new setting for future investigations of cardiomyopathic subjects but, importantly, offers better understanding of the processes and expected physiological transitional responses following cardiac recovery, when the process of gradual weaning of patients from various MCS techniques takes place.

### Study strengths

The strengths of our study include rigorous adherence to the prespecified protocol, short inception period and high levels of data integrity. The enrolled animals were uniform in age and size. All procedures were performed by expert veterinarian anesthesiologists, cardiac human and veterinarian surgeons, perfusionists, cardiologist and intensive care specialist with extensive experience of clinical and research work including MCS and ovine models. The left main coronary artery in sheep supplies blood to almost the entire LV. We directly measured left main coronary flow, arterial and venous oxygen content, which allowed unassumed certainty about the results. To our knowledge, this is the first head-to-head comparative investigation of the impact by the three MCS techniques on myocardial oxygen consumption in normal hearts, utilizing direct measurements of coronary flow, arterio-venous oxygen content and myocardial work.

### Limitations

The limitations of this study include a relatively small sample size dictated by the principles of responsible animal ethics in research, substantial animal to animal variation and the requirement for complex statistical analyses to account for those variations. The possibility of type 2 statistical errors exists. Estimating sample size in advance was not possible due to the absence of available data for calculation. Standardization of MCS application, rather that randomization of interventions was chosen to avoid overcomplication of already difficult statistical interpretation in a relatively small number of animals. The open chest, normal heart ovine model provided findings that although informative, are not immediately translatable to the common clinical practice of closed chest peripheral ECMO for sick hearts. High Impella flow measurements were unreliable for precise estimation of native LV output as were PV loop analyses during high flow settings of all investigated MCS techniques. Some of the haemodynamic responses were inconsistent with those normally seen in MCS management of critical cardiomyopathy such that the findings can only be translated to patients who do not require MCS (e.g. recovered but not weaned) and only with caution. Finally, the study was designed to be performed on normal hearts with normal coronary system. While some may perceive it as a limitation, we believe that such translational study was essential to expand the knowledge and understanding of complex physiological responses to the MCS intervention, since normal human data would be impossible to obtain due to the ethical considerations. Such information is needed to better comprehend responses specific to various pathologies requiring MCS, and to better optimize MCS support in recovering hearts.

## Materials and methods

### Study design

We conducted a prospective, interventional study in adult first cross merino sheep in Sydney Imaging’s Hybrid Operating Theatre at the University of Sydney, Australia. The study was approved as an extension of projects previously approved by the University of Sydney (Australia) Animal Research Ethics Committee (2022/2179) and was conducted at the Charles Perkins Centre, The University of Sydney (Sydney, Australia). The study complied with institutional general guidelines and regulations and ARRIVE guidelines for in-vivo animal experiments. The study protocol was finalized before data collection was initiated. The investigation was performed in accordance with the Helsinki Convention guidelines for humane care of animals.

### Subjects

Seven adult two-year-old Merino first-cross ewes (weight 49.9 ± 1.0 kg) were acclimatized for at least two weeks prior to the procedure and received routine preventative treatment prior to arrival. On the day of the procedure an intravenous catheter (Optiva 18 g (45 mm), Smith Medical, Minneapolis, MN, USA) was inserted into a cephalic vein. and the sheep premedicated intravenously (IV) with a combination of methadone 0.2 mg/kg and midazolam 0.4 mg/kg. Anesthesia was induced by administering propofol IV to effect, to facilitate orotracheal intubation. Anesthesia was maintained with inspired 1–2% isoflurane delivered in an air/oxygen mixture and ketamine at an IV infusion rate of 20 µg/kg/min. Morphine 0.5 mg/kg IV was administered every four hours, or as determined necessary by the anaesthetist. All sheep were mechanically ventilated using volume-controlled intermittent positive pressure ventilation with a positive-end-expiratory pressure of 5 cmH_2_O. A left thoracotomy was performed via the 4th intercostal space and the right atrium and aortic arch cannulated for central ECMO. Following a 15-minute period on cardiopulmonary bypass, an Impella CP (Abiomed, Danvers, MA, USA) was implanted via the left carotid using a Seldinger technique. The correct positioning of the microaxial pump was confirmed under epicardial echocardiographic guidance, as recently described^20^. Epicardial echocardiography excluded significant valvular abnormalities and showed only mild aortic valve incompetence in all sheep following implantation of the Impella and repeatedly during MCS.

Fluid therapy and vasopressor support were used as required to maintain normal blood pressure and cardiac output between separate MCS runs, but not between changes in MCS flows. All experiments were performed with normothermia, and the chest remained open.

At the end of the final study procedure, the sheep were then used for a parallel study of mitral valve interventions prior to euthanasia in accordance with that study protocol.

### Measurements

Fluid filled manometer catheters were placed in the external jugular vein and the aortic arch and continuously recorded (Philips Patient Monitor, IntelliVue MX800 and IntelliSpace Critical Care and Anesthesia (ICCA) software, Philips Medizin Systeme Boeblingen GmbH, Hewlett-Packard-Str.2 71034 Boeblingen, Germany). A straight pressure catheter was positioned in the left atrium and a straight pressure-volume conductance catheter (Millar Inc, Houston, TX) was inserted transapically into the left ventricle with the tip positioned in the ascending aorta for stabilisation. The catheters were calibrated using the Millar Pressure Volume Loop System (MPVS) control software and connected to a data acquisition hardware device (Powerlab 8/35, ADInstruments, Bella Vista, NSW) for reliable data acquisition. A transit-time ultrasonic flow probe (Transonic, Ithaca, NY) was placed around the main pulmonary artery to measure the cardiac output and around the left main coronary artery for direct continuous measurement of coronary artery flow. As the left main coronary artery effectively supplies the entire LV in sheep, this measurement was used to calculate LV oxygen consumption. Oxylite oxygen probes (Oxford Optronics, Adderbury UK) were inserted via the pressure monitoring cannula in the aortic arch and via a cannula in the great cardiac vein or the coronary sinus (hemiazygos vein ligated) for continuous measurement of oxygen partial pressures. All recording devices were linked to a data analysis software program (LabChart, ADInstruments, Bella Vista, NSW) which allowed continuous simultaneous acquisition of all signals. At baseline and each level of all MCS flow, aortic and coronary venous blood samples were obtained and analyzed for oxygen saturation and haemoglobin levels (epoc^®^ Blood Analysis System, Siemens Healthineers, Germany).

Multiple haemodynamic parameters and aortic and coronary sinus oxygen partial pressures and saturations were recorded at the baseline and then at variable settings of the mechanical cardiac support.

### Calculated measuements

Left ventricular native cardiac output (LV CO) = main PA flow – Impella flow (L/minute) (Impella flows were recorded from the Impella controller console).

LV stroke volume (LV SV) calculated as: LV CO/HR (ml/minute).

Total Systemic Blood Flow (TSBF) = PA flow + ECMO flow (ECMO using roller pump console measurement) (L/minute).

Native LV stroke work (LV SW) = LV SV * (SBP-LAP). (mmHg*ml) (a surrogate for the measurement of the pressure volume loop area), and LV SV * (MAP-LAP). (mmHg*ml) (The standard calculation used in clinical practice)^21^.

Systemic vascular resistance (SVR) = 80*(MAP-CVP)/(TSBF). (mmHg*minute/litre)

Arterial elastance (Ea) = 0.9*SBP/(Native LV CO/HR).

LV myocardial oxygen consumption = 1.34*Hb*(SaO_2_-SvO_2_)*coronary flow. (ml/minute)

where Hb is the haemoglobin blood concentration (mg/dl) and SaO_2_ and SvO_2_ are the arterial and venous blood oxygen saturations (%).

### Mechanical circulatory support

Each animal had two separate runs of all three MCS configurations in the following order with flows incrementally increased from zero to maximum achievable, with time allowed for stabilization between the flow increases and between the each MCS run.

### Impella CP

The Impella flow was set at zero and then gradually increased stepwise using the Impella power settings on the Impella console, up to the maximum flow achievable and then returned to zero again. 4–5 stepwise increases in flow were performed in all sheep with 3–5 min allowed for haemodynamic stabilization between each step.

### ECMO

A standard CPB circuit using a roller-pump was converted to a closed circuit for ECMO with right atrial and aortic arch cannulae. ECMO flows were increased stepwise from zero to match those achieved by the Impella, and then returned to zero again with 3–5 min allowed for haemodynamic stabilization between each step.

### ECPELLA

Flows on the V-A ECMO pump and in the Impella were gradually increased in equal amounts until the maximum achievable on one of the devices. The number of steps for the flow increase varied from 3 to 5, with 3 min allowed for the haemodynamic stabilization after each step.

Haemodynamic parameters were recorded, and coronary venous blood samples were taken at zero flow (baseline) and at each level of MCS flow for each device. The experiment for the three MCS was then repeated in its entirety, starting from the baseline for each animal.

### Statistical analyses

Mean values for haemodynamic variables within each sheep were calculated at baseline and for highest MCS flows at each experimental run. Continuous variables expressed as the mean ± standard deviation or the median and interquartile range (IQR) as appropriate.

A mixed-effects linear regression model was fitted to examine the effect of increased device flow on each of the dependent haemodynamic variables, treating sheep as a random effect to account for repeated measurements. The variability in baseline values was accounted for by incorporating random intercepts and slope separately for each animal, which assumes different effect sizes for each animal. The model formula used was hemodynamic_parameter ~ device_flow * device + (experimental_run | sheep)>. The beta coefficients are expressed as the change in parameter unit per litre increase in MCS flow. Bonferroni adjustments were performed for multiple testing where indicated. Adjusted R^2^ was calculated for pre-specified covariates of interest. Additional pre-specified comparisons examining interaction effects of varying hemodynamic parameters were also performed using the mixed-effects linear regression methodology. P value < 0.05 was considered significant.

Finally, in view of the absence of randomisation of the order of MCS, we performed an analysis of baseline values of each parameter, specifically looking at pairwise differences between the baseline values for each pair of Impella vs. ECMO, Impella vs. ECPELLA, and ECMO vs. ECPELLA.

Statistical analyses were performed using R (R Core Team [2021], Vienna, Austria; version 4.3.1), with package < lme4> (version 1.1–34), < MuMIn> (version 1.47.5), < htestClust> (version 0.2.2), < relaimpo> (version 2.2-7), and < afex> (version 1.3-0).

## Conclusions

All MCS techniques increased arterial elastance, but reduced LV myocardial work, coronary arterial flow and LV myocardial oxygen consumption, without significant difference between Impella and ECMO. ECPELLA was more effective in increasing total systemic blood flow and MAP. The overall similarity between the MCS techniques suggests that the more invasive and complex combination of devices (ECPELLA) can only be justified for management of the severe failing heart as the means for decompressing LV. A study investigating the comparative impacts of different regimes and MCS techniques in a cardiomyopathic model is warranted.

## Data Availability

Raw data will be made available on reasonable request to the corresponding author via email: syastrebov@y7mail.com.

## Abbreviations

ECMO: Veno-arterial extracorporeal membrane oxygenation
MCS: Mechanical circulatory support
HR: Heart rate
LV: Left ventricle
RV: Right ventricle
SBP: Systolic blood pressure
DBP: Diastolic blood pressure
MAP: Mean arterial pressure
PP: Pulse pressure
CVP: Central venous pressure
LVEDP: Left ventricular end diastolic pressure
LAP: Left atrial pressure
CO: Cardiac output
SV: Stroke volume
SVR: Systemic vascular resistance
